# Cadmium Activates EGFR/STAT5 Signaling to Overcome Calcium Chelation and Promote Epithelial to Mesenchymal Transition

**DOI:** 10.3390/biom13010116

**Published:** 2023-01-06

**Authors:** Aikaterini Stavrou, Angelica Ortiz, Max Costa

**Affiliations:** Department of Medicine, Division of Environmental Medicine, New York University Grossman School of Medicine, New York, NY 10010, USA

**Keywords:** Cadmium, EGFR, carcinogenesis, metals, STAT5, E-Cadherin, N-Cadherin, Cd, H3.1, EMT, Epithelial-to-Mesenchymal Transition

## Abstract

Cadmium (Cd) is a heavy metal found in cigarette smoke, as well as in air and drinking water due to agricultural and industrial activities, and it poses a health risk to the general population. Prolonged low-dose Cd exposure via inhalation or ingestion causes lung and kidney cancers in humans and in animal models. While high doses of Cd exposure are correlated with the occupational setting and are cytotoxic, low doses of Cd are mainly correlated with exposure in the general population and induce carcinogenesis. The mechanism by which Cd-exposed cells overcome calcium chelation and induce malignant transformation remains unclear. This study examines how cells exposed to low doses of Cd survive loss of E-cadherin cell-cell adhesion via activation of the epidermal growth factor receptor (EGFR) and signal transducer and activator of transcription 5 (STAT5), which work to upregulate genes associated with survival and proliferation. To demonstrate the role of Cd in EGFR/STAT5 activation, we exposed two epithelial cell lines, BEAS-2B and HEK293, to two different doses (0.4 µM and 1.6 µM) of Cadmium chloride hemipentahydrate (CdCl_2_·2.5H_2_O) that are environmentally relevant to levels of Cd found in food and cigarettes for 24 h (hours) and 9 weeks (wks). When comparing cells treated with Cd with control cells, the Cd treated cells exhibited faster proliferation; therefore, we studied activation of EGFR via the STAT5 pathway using immunofluorescence (IF) for protein expression and localization and, in addition, RT-qPCR to examine changes in EGFR/STAT5 inducible genes. Our results showed an increase in EGFR and phosphorylated EGFR (p-EGFR) protein, with 1.6 µM of Cadmium having the highest expression at both 24-hour (hr) and 9-week (wk) exposures. Moreover, the IF analysis also demonstrated an increase of STAT5 and phosphorylated STAT5 (pSTAT5) in both short-term and long-term exposure, with 0.4 µM having the highest expression at 24 h. Finally, via Western blot analysis, we showed that there was a dose-dependent decrease in E-cadherin protein expression and increased N-cadherin in cells treated with low doses of Cd. These data demonstrate that epithelial cells can overcome Cd-mediated toxicity via activation of EGFR pathway to induce cell proliferation and survival and promote epithelial to mesenchymal transition.

## 1. Introduction

Cadmium (Cd), a toxic heavy metal, is categorized as a Group 1 “human carcinogen” by the IARC [1,2]. Naturally occurring Cd is detectable in the air due to forest fires and volcanoes, which can then permeate the water and ground. Anthropogenic cadmium, a result of agricultural and industrial activities, is also detectable in soil and groundwater [1,2,3]. As such, Cd exposure is not limited to industrial workers but can also pose a health risk to the general population. The non-smoking general population is primarily exposed to Cd via the ingestion of contaminated foods on a daily basis; in smokers, the primary route of exposure is via inhalation (1.7 μg per cigarette) [3]. Intake of Cd from food varies per country, as less developed nations do not have declared standards for Cd levels in food and water. For example, food in Japan, such as rice, has high levels of Cd, averaging to 25.5 μg/day, and the population there is more at risk [4,5]. Whereas in the US, according to ATSDR and CDC reports [3], the average geometric estimated mean intake of Cd from food sources by the general population is 18.9 μg/day. On average, daily Cd intake via food ingestion in most countries is approximated to be 0.1–0.4 μg/kg. Moreover, non-smokers have mean blood Cd concentrations of 0.28 μg/L [3]. Thus, the doses of 0.4 μM and 1.6 μM that were used in this study are environmentally relevant to real-life exposure scenarios for the general population via ingestion of Cd-contaminated food. Cd has a long half-life in humans once it is absorbed (7–16 years), so it bioaccumulates over time. Acute exposure to Cd can happen when one travels to countries such as Bangkok, Thailand, and South-East Asia, where they can be exposed to high doses of Cd in the food [3]. Due to the globalization of modern society, travelers must be wary of acute and chronic Cd exposures in the countries they visit. Therefore, examining pathways altered by both acute and chronic exposures to Cd and how these changes affect inflammatory diseases such as carcinogenesis is of great concern.

While Cd is not a mutagen or capable of forming DNA adducts, Cd exposure is often associated with increased oxidative stress and epigenetic changes that then result in transcriptional alterations associated with malignant cell transformation. However, these Cd-mediated epigenetic changes became permanent upon the completion of malignant transformation. The mechanisms activated by Cd to overcome its toxicity and promote cell survival during the epithelial to mesenchymal transition (EMT) remain unclear. EMT is the process by which epithelial cells adopt mesenchymal phenotypes. Cells that undergo EMT are then conferred with cell behavior resembling that of cancer cells. Hallmarks of cancer cells include decreased expression of E-cadherin (E-cad), increased expression of N-cadherin (N-cad), an increase in cell survival and/or proliferation, immortality, and anchorage-independent growth. These changes are often associated with upregulation of transcription factors associated with mesenchymal cells, such as Zinc finger E-box binding homeobox 1 (ZEB1). ZEB1 mediates EMT and promotes tumor invasion and metastasis in cancer cells [6]. In the absence of E-Cad, cancer cells undergo increased activation of the epidermal growth factor receptor (EGFR) [7,8]. EGFR, a receptor tyrosine kinase, is often upregulated in cancers such as non-small-cell lung cancer [9,10,11,12,13,14,15]. Activated EGFR participates in the activation of various cytosolic proteins to affect transcription but can also translocate to the nucleus to alter transcription [10,12,13,14,15,16,17]. While EGFR can localize to the nucleus to affect transcription, this protein does not have a DNA-binding domain [10,13,16,18]. Instead, EGFR interacts with other transcription factors, such as STAT5, to activate gene expression by targeting AT-rich sequences in gene promoters [10,13,16,18]. STAT5 belongs to the STAT family of transcription factors, and it is an oncogenic downstream mediator of the JAK/STAT signaling pathway [19,20,21,22]. STAT5 refers to two highly related genes, STAT5A and STAT5B [19,20,21,22]. This study will demonstrate that during both acute and chronic Cd exposure, cells overcome loss of E-cad function and Cd-mediated toxicity via activation of the EGFR/STAT5 pathway, allowing transcriptional changes that initiate EMT and increase cell proliferation and survival. The Cd doses used in this study were chosen according to epidemiological data available [23] and information obtained by IARC, ATSDR, and the CDC [3,4] in order to be environmentally relevant to non-occupational human exposure.

## 2. Materials and Methods

### 2.1. Cell Culture

Human embryonic kidney cells (HEK293) and human bronchial epithelial cells (BEAS-2B) were purchased from ATCC (Manassas, VA, USA). HEK293 cells were cultured in Dulbecco’s modified Eagle’s medium (DMEM) (Invitrogen, Carlsbad, CA, USA), supplemented with 10% fetal bovine serum (Gibco, ThermoFisher, Waltham, MA, USA), 10 U penicillin/mL, and 10 g streptomycin/mL (ThermoFisher Scientific, Waltham, MA, USA). Cells were maintained in 10 cm tissue culture plates at 37 °C under 5% CO_2_. BEAS-2B cells were cultured in either DMEM (Invitrogen, Carlsbad, CA, USA), supplemented with 10% fetal bovine serum (Gibco, ThermoFisher) for the short term treatments, or 1× Bronchial Epithelial Cell Growth Medium (BEGM) (Lonza, Basel, Switzerland) SingleQuots Supplement Pack (Lonza, Basel, Switzerland) containing 2 mL BPE, 0.5 mL Insulin, 0.50 mL Hydrocortisone, 0.5 mL GA-1000, 0.5 mL Retinoic Acid, 0.5 mL Transferrin, 0.5mL Triiodothyronine, 0.5 mL Epinephrine, and 0.5 mL hEGF for the long term treatments. Cells were maintained in 6-well tissue culture plates at 37 °C under 5% CO_2_. The BEAS-2B cells cultured for 9 weeks in BEGM were maintained in 6-well tissue culture plates coated with a 2:1 mixture of 0.1% gelatin (s006100, Gibco, ThermoFisher) and 0.01 mg/mL (Gibco, ThermoFisher).

### 2.2. Cadmium Treatments

Cadmium chloride hemipentahydrate (CdCl_2_·2.5H_2_O) (Acros Organics, Gael, Belgium) was diluted in Milli-Q water to make a 100 mM stock solution, which was filter sterilized using a 0.22 µm filter before making the 0.4 µM and 1.6 µM dilutions in the culture media. Initial Cd exposure for both doses began once the cells reached 50% confluence. Control samples were also cultured using an equal volume of Milli-Q water diluted in the respective media. Cells were treated for 24 h or 9 weeks. HEK293 cells treated for 9 weeks were starved of fetal bovine serum for 24 h prior to collecting RNA and protein.

### 2.3. Real-Time Quantitative PCR

Following 24 h, cells were washed twice with ice-cold PBS before adding TRIzol reagent (Ambion, Austin, TX, USA) for RNA isolation. For the 9-week treated cells, cells were lifted and counted, then seeded on 6-well plates with their respective condition media and once they reached 80% confluence, total RNA was extracted using Trizol reagent (Ambion, Austin, TX, USA). RNA concentration was determined using NanoDrop 2000/2000c. RNA was used to make cDNA using the Applied Biosystems High-Capacity RNA-to-cDNA kit (ThermoFisher). The resulting product was diluted in nuclease-free water for RT-QPCR using Power SYBR Green PCR master mix (ThermoFisher) and QuantStudio 6 Flex Real-Time PCR (ThermoFisher) Systems. Table 1 contains the details of the RT-QCR primers used in this study. All reactions were performed in triplicate, and RT-QPCR was repeated three times.

### 2.4. Immunofluorescence Assay

Cells were lifted from culture using 1 mM EDTA in PBS, and 5000 cells/well were seeded on 12 mm diameter, 1.5 mm-thick round glass cover slips (Thomas Scientific, Swedesboro, NJ, USA) in a 24-well plate. Cells were cultured in their respective media and treated with Cd until they reached 50% confluence before fixing with ice-cold methanol for analysis. The cells were rehydrated with PBS and permeabilized with 0.2% Triton in PBS before blocking with 5% goat serum in PBS (Gibco, ThermoFisher) for one hour. The cells were then incubated overnight at 4 °C with the primary antibodies (Table 2). After incubation with the primary antibodies, cells were washed with 0.001% Triton-X100 in PBS and incubated for 1 h at room temperature with the secondary fluorescent antibodies, Alexa Fluor 488 (goat anti-Mouse IgG, IgM (H + L), 1:500, ThermoFisher), and TRITC (Goat anti-Rabbit IgG (H + L), 1:200, ThermoFisher). The coverslips with the fluorescently labeled cells were placed onto glass slides with ProLong Gold Antifade Mountant with 4′,6-diamidino-2-phenylindole (DAPI) (ThermoFisher) mounting solution, and images were captured using a Zeiss 880 Laser Scanning Confocal microscope (NYULH microscopy core, NY). All treatments were done in triplicate. Quantification of mean fluorescent intensity was performed as previously described using Fiji ImageJ [24], and data were expressed as fold change.

### 2.5. Western Blot

Cells treated for 6 weeks with 1.6 μM and 0.4 μM Cd were seeded in a 6-well plate in duplicates with their respective culture media. Once cells reached 80% confluence, protein was collected in RIPA lysis buffer. Protein concentration was quantified using the Pierce BCA Protein Assay Kit (ThermoFisher). Protein samples were resolved in 4–15% Mini-PROTEAN TGX™ Precast Protein Gels (BioRad, Hercules, CA, USA) and then transferred to PVDF membrane. Following transfer, the membrane was blocked with 5% bovine serum albumin (BSA, Sigma, St. Louis, MO, USA) in PBS for 1 h at room temperature. The membrane was then incubated overnight with the primary antibody (Table 3) at 4 °C. The next day, the membrane was washed with 1X Tris-buffered saline tween-20 (0.1%, ThermoFisher) (TBST) and then incubated with the secondary antibody (Table 3) for 1 h at room temperature. Western blot analysis was conducted using a chemiluminescent substrate (ECL) kit (ThermoFisher) for 1 min at room temperature and X-ray film exposure. Western blot signals were quantified using ImageJ.

### 2.6. Statistical Analysis

All results are presented as means ± SD of three independent experiments. Significant differences between groups were evaluated using a one-way ANOVA multiple comparison using GraphPad Prism 9 software (GraphPad, San Diego, CA, USA). A *p*-value < 0.05 was considered statistically significant in all cases.

## 3. Results

### 3.1. Epithelial Cells Overcome Cadmium-Mediated Loss of E-Cadherin by Increasing N-Cadherin

**Figure 1:**
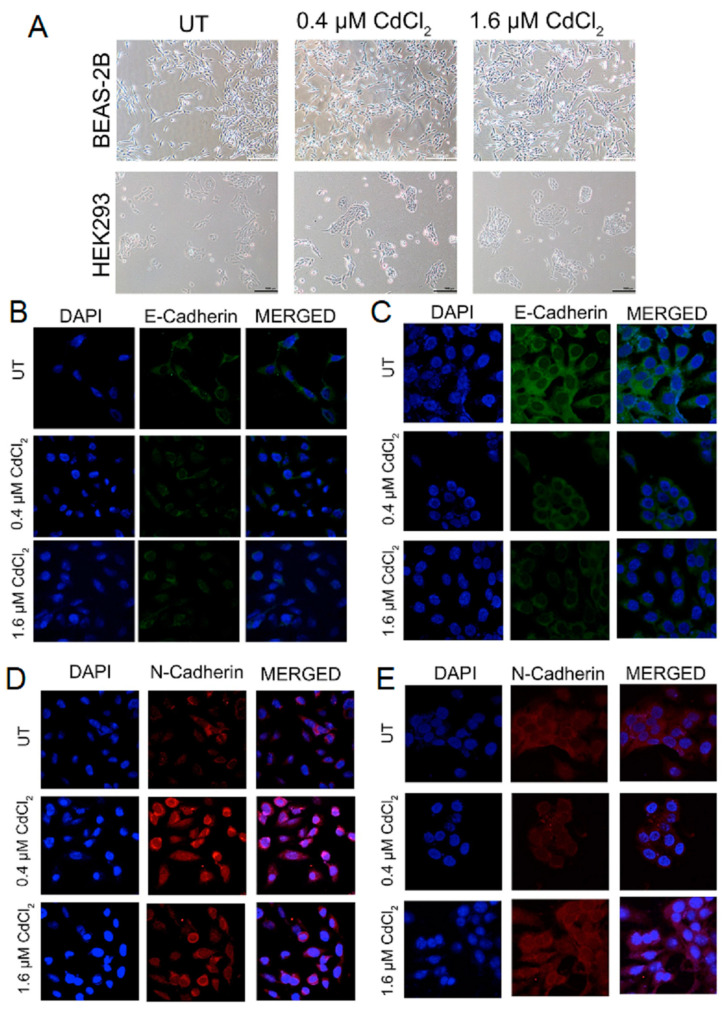
Low doses of Cadmium alter cell adhesion through changes in cadherin expression. (**A**) Representative bright-field images of BEAS-2B and HEK293 cells treated with 0.4 μM or 1.6 μM Cd for 24 h. This figure depicts morphological differentiations observed after 24 h of treatment under 10× magnification (scale bar, 1000 μm). (**B**,**C**) Representative images from the IF assays on BEAS-2B (**B**) and HEK293 (**C**) cells treated with 0.4 μM or 1.6 μM Cd for 24 h. Blue color = DAPI-stained nuclei; green color = E-cadherin-AlexaFluor488. (**D**,**E**) IF assay on BEAS-2B (**D**) and HEK293 (**E**) cells treated with 0.4 μM or 1.6 μM Cd for 24 h. Blue color = DAPI-stained nuclei; red color = N-cadherin-TRITC. Confocal microscopy pictures were taken under 63x magnification (UT = untreated controls).

Similar to EDTA, Cd functions as a chelating agent, removing calcium ions from calcium-dependent proteins such as E-cad. Removal of calcium from E-cad results in loss of adhesion and subsequent loss of E-cad at the cell surface. Following the loss of E-cad, normal cells undergo anoikis. We expected to observe cells detach from the tissue culture plate and dead cells in the culture media. However, 24 h after changing the media to equalize the number of seeded cells, we found that cells in 0.4 and 1.6 µM Cd were all attached (Figure 1A). Moreover, all wells with Cd-containing media seemed to be more confluent with cells (Figure 1A). This suggested that this low dose of Cd exposure after 24 h was not sufficient to reduce E-cad protein at the cell surface. Therefore, we set out to examine changes in E-cad on the cell surface. The cells were again seeded in equal numbers (BEAS-2B and HEK293) onto glass cover slips and cultured with normal media (UT) or media with low doses of Cd (0.4 or 1.6 µM) for 24 h. Immunofluorescence (IF) analysis demonstrated that E-cadherin levels were decreased in BEAS-2B cells at both doses (Figure 1B and Figure S1A). This loss of E-cad was confirmed using Western blot analysis (Figure S1E). In HEK293 cells, cell surface E-cad levels were decreased only at 1.6 µM of Cd (Figure 1C and Figure S1B). However, Western blot analysis demonstrated that these low doses of Cd did not affect total E-cad protein levels in HEK293 cells after 24 h (Figure S1F). Considering that 24 h exposure to Cd did not result in anoikis in BEAS-2B cells or in the 1.6 µM treated HEK293 cells, we set out to examine if this time point was sufficient to induce cadherin switching resulting in changes in the protein level of N-cadherin (N-cad). Interestingly, IF analysis demonstrated that 0.4 µM of Cd increased N-cad the most when compared to the untreated condition in BEAS-2B cells (Figure 1D and Figure S1C). However, HEK293 cells exhibited the opposite effect, with only the 1.6 μM condition having a significant increase in N-cadherin expression (Figure 1E and Figure S1D). Western blot analysis also demonstrated that BEAS-2B cells treated with both doses of Cd for 24 h expressed more N-cad total protein compared to untreated cells (Figure S1E). Western blot of HEK293 samples demonstrated the same N-cad protein pattern as the IF analysis (Figure S1F). While the cadherin switching explained how the epithelial cells survived the loss of E-cad and maintained cell adhesion to the tissue culture plate to survive, we did not observe similar compensation for adhesion and survival in the cells treated with 0.4 µM of Cd. Therefore, we examined the status of EGFR, which is often activated in the absence of E-cad-mediated adhesion.

### 3.2. Low Doses of Cadmium Increased Phosphorylation of EGFR and STAT5 after 24 h

**Figure 2:**
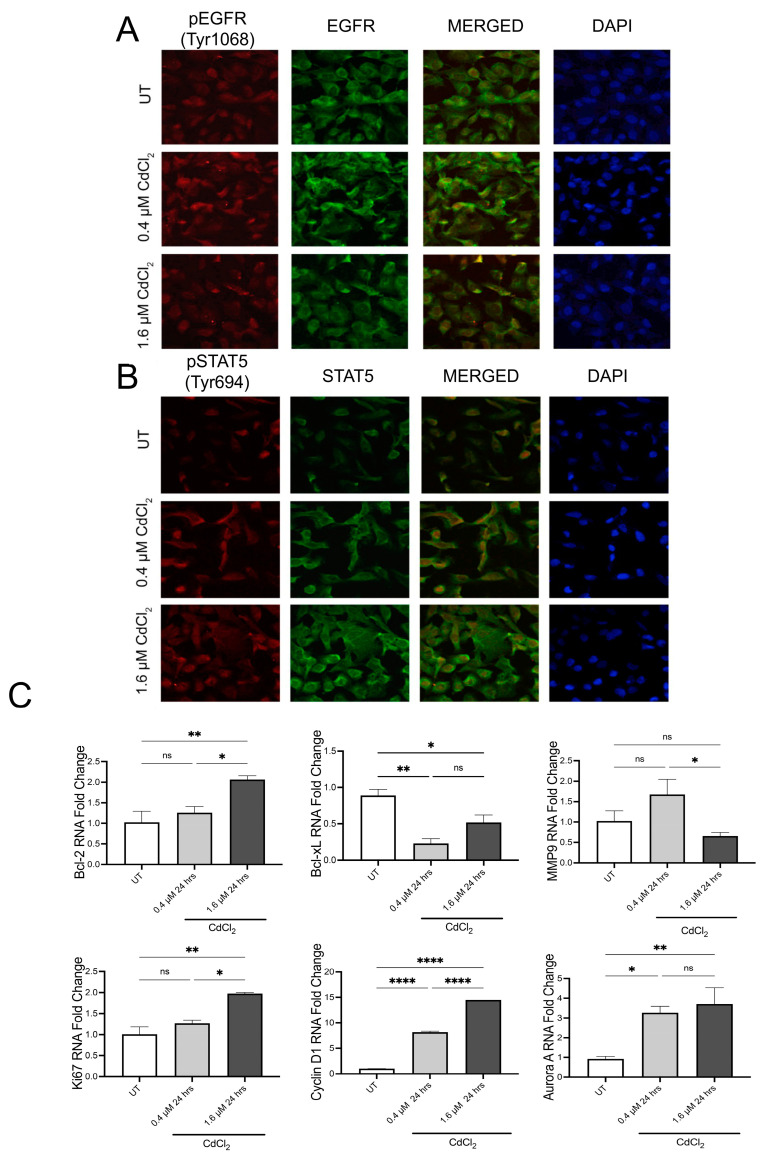
Low doses of Cd activate EGFR/STAT5 signaling in BEAS-2B cells. (**A**) Representative fluorescent images of the IF assay on BEAS-2B cells treated with 0.4 μM or 1.6 μM Cd for 24 h in DMEM media to examine changes in EGFR phosphorylation. Green color = EGFR-AlexaFluor488 (total EGFR); red color = pEGFR (Tyr1068)-TRITC. Confocal microscopy pictures were taken under 63× magnification. (**B**) Representative fluorescent images of the IF assay on BEAS-2B cells treated with 0.4 μM or 1.6 μM Cd for 24 h in DMEM media to examine changes in STAT5 phosphorylation. Green color = STAT5-AlexaFluor488 (total STAT5); red color = pSTAT5 (Tyr694)-TRITC. Confocal microscopy pictures were taken under 63× magnification. (**C**) mRNA levels of proliferation markers and downstream components of the EGFR-STAT5 pathway, from BEAS-2B cells treated with 0.4 μM or 1.6 μM Cd for 24 h, expressed as fold change. Values shown are means ± SE. * *p* < 0.05, ** *p* < 0.01, **** *p* < 0.0001 vs. UT (Untreated controls).

**Figure 3:**
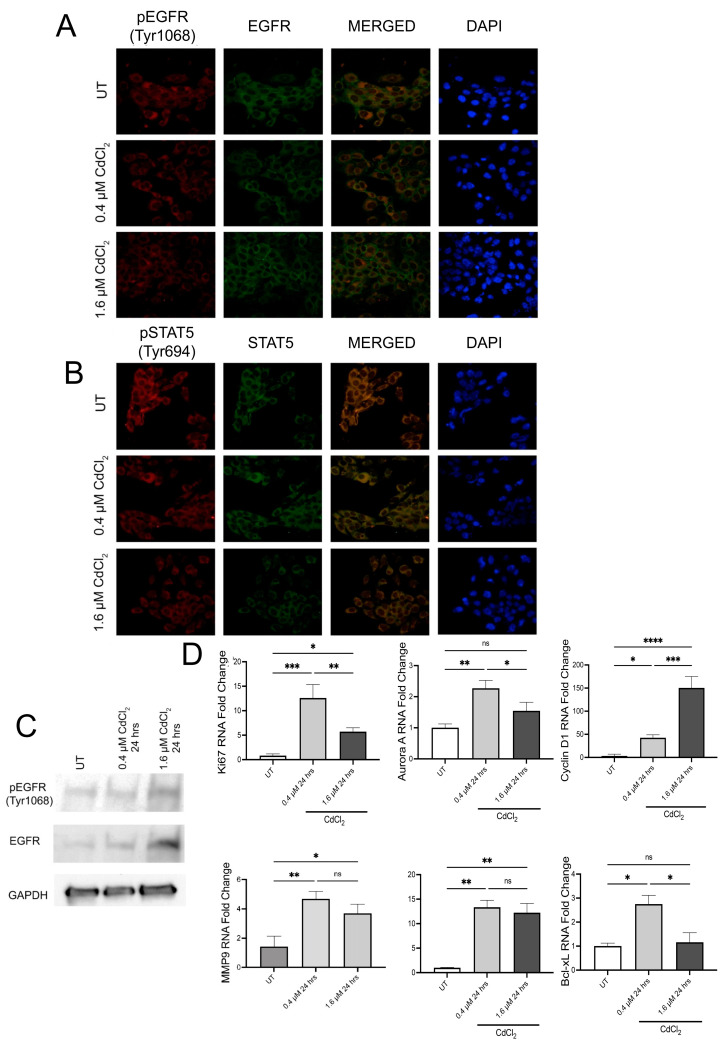
Low doses of Cd increase phosphorylation/activation of the EGFR/STAT5 pathways in HEK293 cells. (**A**) Representative fluorescent images of the IF assay on HEK293 cells treated with 0.4 μM or 1.6 μM Cd for 24 h in DMEM media to examine changes in EGFR phosphorylation. Green color = EGFR-AlexaFluor488 (total EGFR); red color = pEGFR (Tyr1068)-TRITC. Confocal microscopy pictures were taken under 63× magnification. (**B**) Representative fluorescent images of the IF assay on HEK293 cells treated with 0.4 μM or 1.6 μM Cd for 24 h in DMEM media to examine changes in STAT5 phosphorylation. Green color = STAT5-AlexaFluor488 (total STAT5); red color = pSTAT5 (Tyr694)-TRITC. Confocal microscopy pictures were taken under 63× magnification. (**C**) Western blot analysis of EGFR and p-EGFR (Tyr1068) from protein samples derived from HEK293 cells treated with 0.4 μM or 1.6 μM Cd in DMEM for 24 h. (**D**) mRNA levels of proliferation markers and downstream components of the EGFR-STAT5 pathway, from EHK293 cells treated with 0.4 μM or 1.6 μM Cd for 24 h, expressed as fold change. Values shown are means ± SE. * *p* < 0.05, ** *p* < 0.01, *** *p* < 0.001, **** *p* < 0.0001 vs. UT (untreated controls).

IF analysis of BEAS-2B cells demonstrated that after 24 h, exposure to both 0.4 µM and 1.6 µM of Cd increased the phosphorylation of tyrosine 1068 on EGFR, the site of EGFR activation (Figure 2A and Figure S2A). Upon merging the total EGFR and pEGFR signals in the cells, we observed elevated levels of pEGFR in the nuclear space in cells treated with both doses of Cd (Figure 2A and Figure S2A). Activated EGFR can then increase STAT5 directly or through Src to induce transcriptional changes. Therefore, we used IF analysis to examine changes in STAT5 phosphorylation after 24 h exposure to 0.4 and 1.6 µM of Cd. Both doses of 0.4 µM and 1.6 µM Cd increased the phosphorylation of tyrosine 694 of STAT5 in BEAS-2B (Figure 2B and Figure S2B). Following DAPI staining, we also observed that pSTAT5 localized to the nucleus as observed with pEGFR (Figure 2B). Nuclear localization of both is associated with the transcription of genes associated with EMT. We, therefore, set out to examine changes in transcription of key EGFR/STAT5 target genes. Using QPCR analysis, we found that after 24 h in 0.4 µM of Cd, BEAS-2B cells downregulate Bcl-xL but upregulate Cyclin D1 and Aurora Kinase A (Figure 2C). Interestingly, BEAS-2B cells treated with 1.6 µM of Cd upregulate Bcl-2, Ki67, Cyclin D1, and Aurora Kinase A (Figure 2C), suggesting a difference in gene transcription is dose-dependent. To verify these effects of low doses of Cd after 24 h exposure on epithelial cells, the semi-differentiated epithelial cell line HEK293 was used. As observed in BEAS-2B, HEK293 cells exposed to 0.4 µM and 1.6 µM of Cd demonstrated increased phosphorylation of tyrosine 1068 on EGFR using IF analysis (Figure 3A and Figure S2C). Western blot analysis demonstrated that HEK293 cells exposed for 24 h to both low doses of Cd increased pEGFR compared to UT HEK293 cells (Figure 3C and Figure S2E). Interestingly, HEK293 cells exposed to 0.4 µM of Cd exhibited increased pSTAT5 compared to UT HEK293 cells. However, HEK293 cells exposed to 1.6 µM of Cd for 24 h demonstrated a significant decrease in pSTAT5 after 24 h compared to UT, suggesting that at this dose these cells are adversely affected or required a different time point (Figure 3B and Figure S2D). QPCR analysis of HEK293 cells treated with 0.4 µM of Cd for 24 h demonstrated upregulation of the EGFR/STAT5 gene targets Ki67, Aurora kinase A, Cyclin D1, MMP9, Bcl-2, and Bcl-xL (Figure 3D). Interestingly, HEK293 cells treated with 1.6 µM of Cd for 24 h exhibited upregulation of Ki67, Cyclin D1, MMP9, and Bcl-2 (Figure 3D). This data suggests that Cd affects EGFR/STAT5 transcription in HEK293 in a dose dependent manner after 24 h.

### 3.3. Cells Undergoing Chronic Exposure to Low Doses of Cadmium Maintain Cadherin Switch

**Figure 4:**
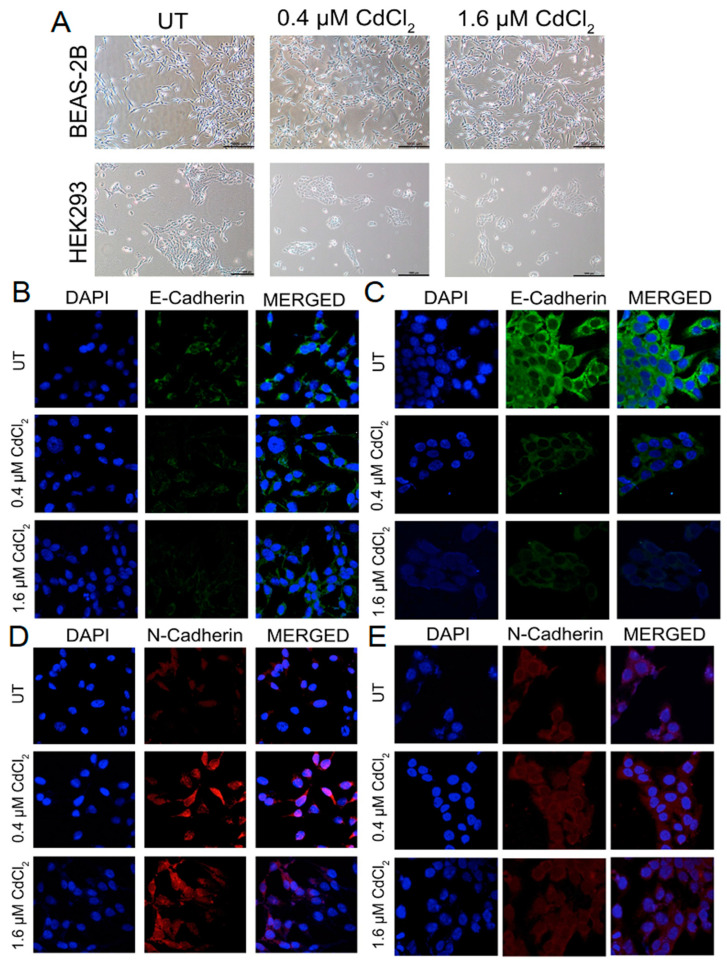
Chronic exposure to low doses of Cd alters cell adhesion via cadherin switching. (**A**) Representative brightfield images of BEAS-2B and HEK293 cells treated either with 0.4 μM or 1.6 μM Cd for 9 weeks in their respective media. This figure depicts morphological differentiations observed after 9 weeks of treatment under 10× magnification (scale bar, 1000 μm). (**B**,**C**) Representative images from IF assays on BEAS-2B (**B**) and HEK293 (**C**) cells treated with 0.4 μM or 1.6 μM Cd for 9 weeks. Blue color = DAPI-stained nuclei; green color = E-cadherin-AlexaFluor488. (**D**,**E**) IF assay on BEAS-2B (**D**) and HEK293 (**E**) cells treated with 0.4 μM or 1.6 μM Cd for 9 weeks. Blue color = DAPI-stained nuclei; red color = N-cadherin-TRITC. Confocal microscopy pictures were taken under 63× magnification (UT = untreated controls).

After 9 weeks in 0.4 and 1.6 µM of Cd, brightfield images demonstrated that both BEAS-2B and HEK293 cells continued to adhere to their culture plates (Figure 4A). Moreover, the Cd-treated cells formed tight cell-cell clusters compared to UT cells (Figure 4A). Moreover, during the passage of cells, we experienced difficulty lifting cells off the plate as well as breaking cell clusters even after they were lifted. This observed effect may be the result of changes in cadherin expression, and therefore, we seeded cells in an equal number onto glass coverslips to examine changes in E-cad and N-cad in BEAS-2B and HEK293 cells. IF analysis demonstrated that after 9 weeks, cells treated with both doses of Cd exhibited significantly decreased E-cad levels (Figure 4B,C and Figure S3A,B). BEAS-2B cells expressed more N-cad after 9 weeks in the presence of 0.4 and 1.6 µM of Cd compared to untreated BEAS-2B cells (Figure 4D and Figure S3C). After 9 weeks, only HEK293 cells treated with the 1.6 µM dose of Cd exhibited increased N-cad compared to untreated HEK293 (Figure 4E and Figure S3D). Considering the sustained cadherin switch, we then examined the status of EGFR and STAT5 and how this cadherin switch may affect EMT.

### 3.4. Chronic Exposure to Cadmium Sustained EGFR/STAT5 Signaling

**Figure 5:**
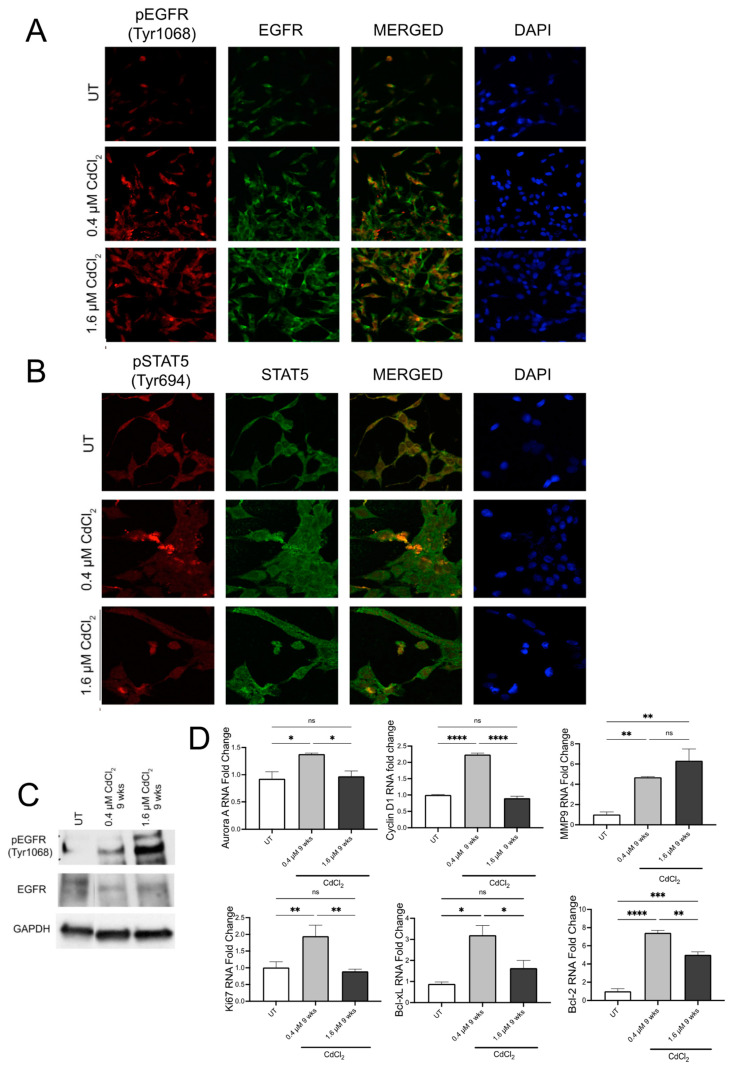
Low doses of Cd increase phosphorylation of EGFR and/or STAT5 but do not activate the EGFR/STAT5 signaling pathway in BEAS-2B cells. (**A**) Representative images from the IF assay on BEAS-2B cells treated with 0.4 μM or 1.6 μM Cd for 9 weeks in BEGM media. Green color = EGFR-AlexaFluor488 (total EGFR); red color = pEGFR (Tyr1068)-TRITC. Confocal microscopy pictures were taken under 63× magnification. (**B**) Representative images from the IF assay on BEAS-2B cells treated with 0.4 μM or 1.6 μM Cd for 9 weeks in BEGM media. Green color = STAT5-AlexaFluor488 (total STAT5); red color = pSTAT5 (Tyr694)-TRITC. Confocal microscopy pictures were taken under 63× magnification. (**C**) Western blot analysis of EGFR and p-EGFR (Tyr1068) from protein samples derived from BEAS-2B 24 cells treated with 0.4 μM or 1.6 μM Cd in BEGM for 9 weeks. (**D**) mRNA levels of proliferation markers and downstream components of the EGFR-STAT5 pathway, from 9-week-old treated BEAS-2B cells with 0.4 μM or 1.6 μM Cd, expressed as fold change. Values shown are means ± SE. * *p* < 0.05, ** *p* < 0.01, *** *p* < 0.001, **** *p* < 0.0001 vs. UT (untreated controls).

**Figure 6:**
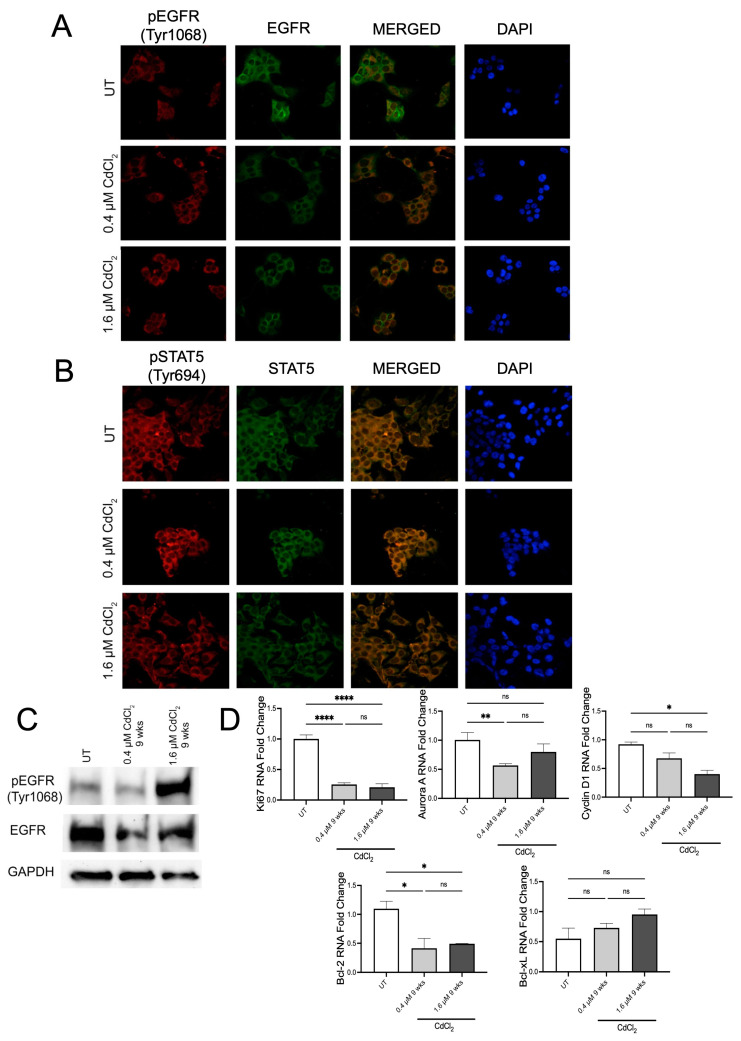
Low doses of Cd increase phosphorylation of EGFR and/or STAT5 but do not activate the EGFR/STAT5 signaling pathway in HEK293 cells. (**A**) Representative images of the IF assay on HEK293 cells treated with 0.4 μM or 1.6 μM Cd for 9 weeks in DMEM media. Green color = EGFR-AlexaFluor488 (total EGFR); red color = pEGFR (Tyr1068)-TRITC. Confocal microscopy pictures were taken under 63× magnification. (**B**) Representative images of the IF assay on HEK293 cells treated with 0.4 μM or 1.6 μM Cd for 9 weeks in DMEM media. Green color = STAT5-AlexaFluor488 (total STAT5); red color = pSTAT5 (Tyr694)-TRITC. Confocal microscopy pictures were taken under 63× magnification. (**C**) Western blot analysis of EGFR and pEGFR (Tyr1068) from protein samples derived from HEK293 cells treated with 0.4 μM or 1.6 μM Cd in DMEM for 9 weeks. (**D**) mRNA levels of proliferation markers and downstream components of the EGFR-STAT5 pathway, from 9-week-treated HEK293 cells with 0.4 μM or 1.6 μM Cd, expressed as fold change. Values shown are means ± SE. * *p* < 0.05, ** *p* < 0.01, **** *p* < 0.0001 vs. UT (untreated controls).

Immunofluorescence analysis demonstrated that after 9 weeks in 1.6 µM of Cd BEAS-2B cells, there was a significant increase in pEGFR when compared to the untreated cells. In the 0.4 μM condition, there was a significant increase in total EGFR. Thus, even though the amount of pEGFR was also increased, the ratio of pEGFR over total EGFR was not statistically increased enough when compared to the untreated cells (Figure 5A and Figure S4A,G). Western blot analysis showed an increase in pEGFR as well (Figure 5C and Figure S4B). Using both IF and Western blot analysis, we found that BEAS-2B cells treated with 1.6 µM of Cd for 9 weeks demonstrated a significant increase in pEGFR (Figure 5A,C and Figure S4A,B). Interestingly, after 9 weeks in 0.4 µM of Cd, BEAS-2B cells had increased pSTAT5 compared to untreated cells, but cells grown in 1.6 µM of Cd for 9 weeks did not increase pSTAT5 compared to untreated (Figure 5B and Figure S4C). However, IF analysis still demonstrated that cells grown in both doses of Cd still exhibited increased pEGFR and pSTAT5 in the nuclear space (when using DAPI as reference) (Figure 5A,B). To demonstrate that cells undergoing chronic exposure to both doses of Cd maintained elevated EGFR/STAT5-mediated transcription, we used QPCR to compare the expression of genes associated with EGFR-STAT5 transcription. QPCR analysis demonstrated that BEAS-2B cells treated with 0.4 µM of Cd for 9 weeks upregulated Aurora kinase A, Cyclin D1, MMP9, Ki67, Bcl-XL, and Bcl-2. BEAS-2B cells treated with 1.6 µM of Cd for 9 weeks upregulated MMP9 and Bcl-2, but not the other genes tested. Analysis of several IF images showed that HEK293 cells showed increased pEGFR after 9 weeks in 0.4 and 1.6 µM of Cd (Figure 6A and Figure S4D). Western blot analysis only showed a significant increase in pEGFR in cells treated with 1.6 µM of Cd for 9 weeks (Figure 6C). IF analysis demonstrated an increase in pSTAT5 in HEK293 cells treated with 0.4 µM of Cd for 9 weeks but not with 1.6 µM of Cd for 9 weeks (Figure 6B and Figure S4F). This suggested that after 9 weeks with both doses of Cd, HEK293 cells do not have active EGFR/STAT5 signaling. This was confirmed with qPCR analysis, demonstrating no increase in expression of EGFR/STAT5 target genes (Figure 6D). Together, these data suggested that after 9 weeks in low doses of Cd, these epithelial cells would express markers of EMT and demonstrate anchorage-independent growth due to independence of EGFR/STAT5.

### 3.5. Chronic Exposure to Low Doses of Cadmium Induces EMT

**Figure 7:**
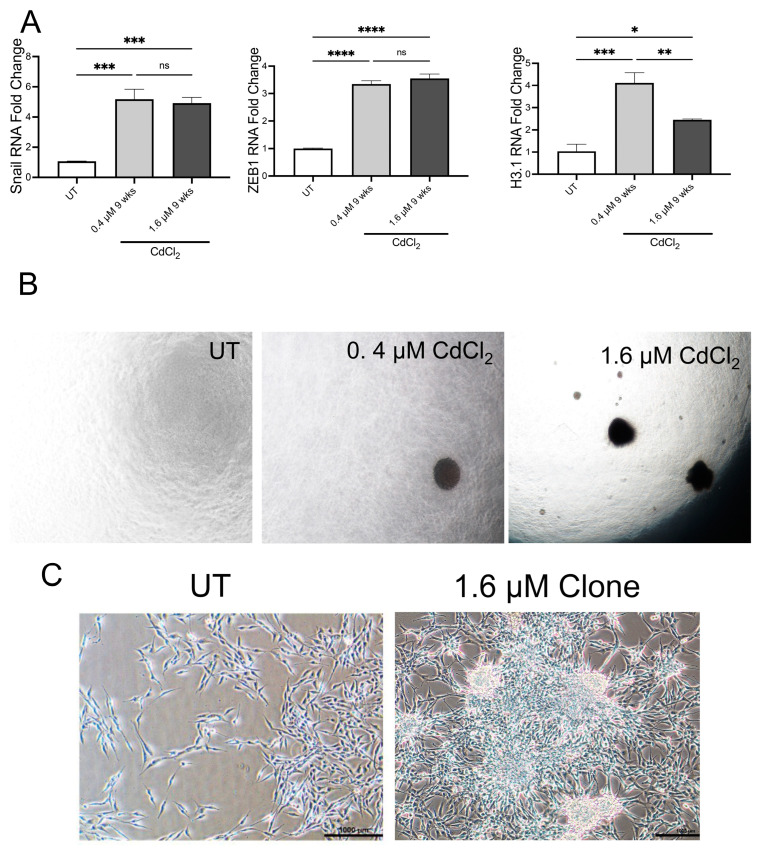
Chronic exposure to low doses of Cd induces EMT in BEAS-2B cells. (**A**) mRNA levels of EMT markers from 9-week-old treated BEAS-2B cells with 0.4 μM or 1.6 μM Cd, expressed as fold change. Values shown are means ± SE. * *p* < 0.05, ** *p* < 0.01, *** *p* < 0.001, **** *p* < 0.0001 vs. UT (untreated controls). (**B**) Representative bright-field images depicting the clones formed on a soft agar assay to assess anchorage-independent growth after exposing BEAS-2B cells to 0.4 μM or 1.6 μM Cd for 9 weeks. Pictures were taken under 4× magnification. (**C**) Representative bright-field images of BEAS-2B-transformed cells (cells from the BEAS-2B clone, transformed with 1.6 μM Cd for 9 weeks) in DMEM media. This figure depicts morphological differentiations observed on the Cd-transformed cells under 10× magnification (scale bar, 1000 μm).

Using QPCR analysis, we examine transcriptional changes associated with metal carcinogenesis. After 9 weeks with both doses of Cd, BEAS-2B cells upregulate Snail, ZEB1, and H3.1 (Figure 7A). These data suggested that after 9 weeks, the Cd-treated cells had adopted sufficient transcriptional and proteomic changes to demonstrate a phenotype associated with cancer malignancy: anchorage-independent growth. Three weeks after seeding the 9-week-old treated and untreated cells into soft agar, the cells treated with both 0.4 and 1.6 µM of Cd generated colonies in soft agar (Figure 7B). A colony from one of the 1.6 µM of Cd-treated wells was isolated from the soft agar and grown on tissue culture plates. The cells that grew from that colony were very different from the untreated BEAS-2B cells (Figure 7C). HEK293 cells exposed to 0.4 µM of Cd began a significant upregulation of Snail and H3.1 after 24 h, but BEAS-2B did not upregulate these markers as significantly (Figure S5A,C). HEK293 cells treated with 1.6 µM of Cd for 24 h showed upregulation in ZEB1 and H3.1 (Figure S5A). These data suggest HEK293 is more sensitive to Cd-mediated transformation compared to BEAS-2B and that the dose of Cd can affect transcriptional changes to induce EMT. Indeed, after 4 weeks rather than 9 weeks, the HEK293 cells treated with low doses of Cd had adopted anchorage-independent growth (Figure S5B), which coincides with the early upregulation of the EMT markers previously observed.

## 4. Discussion

This study reveals a novel mechanism by which low doses of Cd activate EGFR/STAT5 signaling to overcome loss of E-cad-mediated adhesion and induce transcriptional changes that promote cell proliferation and EMT. As part of the EMT process, we observed that N-cadherin was activated, and E-cadherin expression was decreased in just 24 h following exposure to 1.6 μM of Cd. Even though the exact mechanism by which Cd upregulates N-cadherin was not explored in this study, we hypothesize that it is the result of EGFR/STAT5 pathway activation. STAT5 activation has been previously associated with the increase of EMT markers [25]. Previous studies demonstrated that chronic exposure to low doses of Cd induces transformation of BEAS-2B [26,27]. Epigenetic changes associated with malignant transformation were not observed until the BEAS-2B cells were treated for 20 weeks with 2.0 µM of Cd [27]. However, our studies show that at lower doses (i.e., 0.4 µM and 1.6 µM of Cd), both BEAS-2B and HEK293 activate the EGFR/STAT5 pathway after 24 h to begin changes in transcription of genes associated with increased cell proliferation, increased cell survival, and decreased cell apoptosis. Increased EGFR and STAT5 are observed in malignant cancers, and upregulation of their targets is often associated with cancer progression [9,10,15,16,17,21,28]. Therefore, our results showing that low doses of Cd activate oncogenic signaling after 24 h demonstrate how even acute exposures could prove detrimental to the integrity of the epithelial barrier. Furthermore, our results demonstrate that maintaining EGFR/STAT5 signaling ultimately induced elevated expression of SNAIL and ZEB1, often called master regulators of EMT [6,29,30,31,32]. Loss of E-Cad is associated with upregulation of these EMT regulators, and their upregulation promotes the sustained downregulation of E-Cad expression [6,29,30,31,32]. Upregulation of ZEB1 is of particular interest, as increased ZEB1 expression is associated with acquired resistance to EGFR inhibitors [33,34]. Our data demonstrated that after 9 weeks in Cd, BEAS-2B cells could still demonstrate increased EGFR phosphorylation but did not demonstrate increased EGFR/STAT5 signaling compared to untreated cells, i.e., no changes in transcription of EGFR/STAT5 gene targets. This observation suggested that once ZEB1 is upregulated and EMT is complete, the cells no longer rely on EGFR/STAT5 signaling. Therefore, these Cd-transformed cells would be resistant to EGFR inhibitors. Interestingly, we also found that low doses of Cd increase transcription of H3.1 in both BEAS-2B and HEK293 cells, which can result in displacement of the canonical H3.3 histone to promote transcriptional changes associated with EMT [35]. Transcriptional changes induced by upregulation of SNAIL, ZEB1, and H3.1 together likely contribute to the loss of Cd-mediated activation of EGFR/STAT5. Both BEAS-2B and HEK293 cells had increased transcription of SNAIL, ZEB1, and H3.1 at the 9-week time point; however, only HEK293 had a significantly statistical change in the transcription of these genes at the 24-h time point. These observed effects of low doses of Cd on the HEK293 cell line suggest that semi-differentiated epithelial cells or stem cells in epithelial tissues are more susceptible to Cd-mediated EGFR-STAT5 activation to facilitate EMT. Together, the data of this study show that epithelial cells of different differentiation statuses can activate pro-proliferative/anti-apoptotic pathways at low doses of Cd after just 24 h and that continued exposure may increase susceptibility to the possible development of cancer. Finally, as mentioned earlier, the mechanism by which Cd induces carcinogenesis is still unclear. Thus, this is one of the first studies to demonstrate the activation of a novel mechanism that is involved in low-dose Cd-induced carcinogenesis in its very early stages.

## Figures and Tables

**Table 1:** Primers used in this study for RT-qPCR analysis.

Gene	Forward Prime (5′ → 3′)	Reverse Primer (5′ → 3′)
*Bcl-2*	ATCGCCCTGTGGATGACTGAG	CGCCAGGAGAAATCAAACAGAGG
*Bcl-xL*	CTGAATCGGAGATGGAGACC	TGGGATGTCAGGTCACTGAA
*MMP9*	ATCCAGTTTGGTGTCGCGGAGC	GAAGGGGAAGACGCACAGCT
*AURKA*	GGAGAGCTTAAAATTGCAGATTTT	GCTCCAGAGATCCTTCTCAT
*Cyclin-D1*	CTTCCTCTCCAAAATGCCAG	AGAGATGGAAGGGGGAAAGA
*Ki67*	GAGGTGTGCAGAAAATCCAAA	CTGTCCCTATGACTTCTGGTTGT
*ZEB1*	AGCAGTGAAAGAGAAGGGAATGC	GGTCCTCTTCAGGTGCCTCAG
*SNAIL*	ACTGCAACAAGGAATACCTCAG	GCACTGGTACTTCTTGACATCTG
*H3.1*	ACGCCAAGCGGGTGACTAT	TCTCGCCGCGGATACG
*GAPDH*	AGGGCTGCTTTTAACTCTGGT	CCCCACTTGATTTTGGAGGGA

**Table 2:** Antibodies used in this study for IF analysis.

Primary Antibody	Host Species Species	Dilution
EGFR (Santa Cruz Biotechnology, Dallas, TX)	Mouse	1:100
p-EGFR (Tyr1068) (Cell Signaling, Danvers, MA)	Rabbit	1:100
STAT5 (Santa Cruz Biotechnology, Dallas, TX)	Mouse	1:100
p-STAT5 (Tyr694) (Cell Signaling, Danvers, MA)	Rabbit	1:100
N-Cadherin (Cell Signaling, Danvers, MA)	Rabbit	1:100
E-Cadherin (Cell Signaling, Danvers, MA)	Mouse	1:100

**Table 3:** Antibodies used in this study for Western Blot analysis.

Primary Antibody	Host Species Species	Dilution	Secondary Antibody	Dilution
*N-Cadherin* (*Cell signaling*, *Danvers*, *MA*)	Rabbit	1:1000	Goat anti-Rabbit IgG HRP-linked	1:2000
*E-Cadherin* (*Cell signaling*, *Danvers*, *MA*)	Mouse	1:1000	Horse anti-Mouse IgG HRP-linked	1:1000
*EGFR* (*Santa Cruz Biotechnology*, *Dallas*, *TX*)	Mouse	1:1000	Goat anti-Rabbit IgG HRP-linked	1:2000
p-EGFR (Tyr1068) (Cell Signaling, Danvers, MA)	Rabbit	1:1000	Goat anti-Rabbit IgG HRP-linked	1:1000
*GAPDH* (*Proteintech*, *Rosemont*, *IL*)	Mouse	1:10,000	Horse anti-Mouse IgG HRP-linked	1:2000

## Data Availability

Not applicable.

## Supplementary Materials

The following supporting information can be downloaded at: https://www.mdpi.com/article/10.3390/biom13010116/s1, Figure S1: Low doses of Cadmium alter expression in cadherin after 24 h in epithelial cells; Figure S2: Low doses of Cd increase activation of EGFR/STAT5 signaling on both BEAS-2B and HEK293 cells; Figure S3: Low doses of Cadmium alter cadherin expression; Figure S4: Low doses of Cd increase phosphorylation of EGFR and/or STAT5 but do not activate the EGFR/STAT5 signaling pathway in both BEAS-2B and HEK293 cells; Figure S5: Low doses of Cd induce EMT much sooner in HEK293 cells compared to BEAS-2B cells.
