# Supplementary material for: Cadmium Activates EGFR/STAT5 Signaling to Overcome Calcium Chelation and Promote Epithelial to Mesenchymal Transition
Source: Biomolecules. 2023 Jan 6;13(1):116. doi: 10.3390/biom13010116 (PMC9855692; doi:10.3390/biom13010116)
Supplement: Supplementary file 1 [file biomolecules-13-00116-s001.zip › biomolecules-2000992-supplementary.pdf]

Supplementary Materials

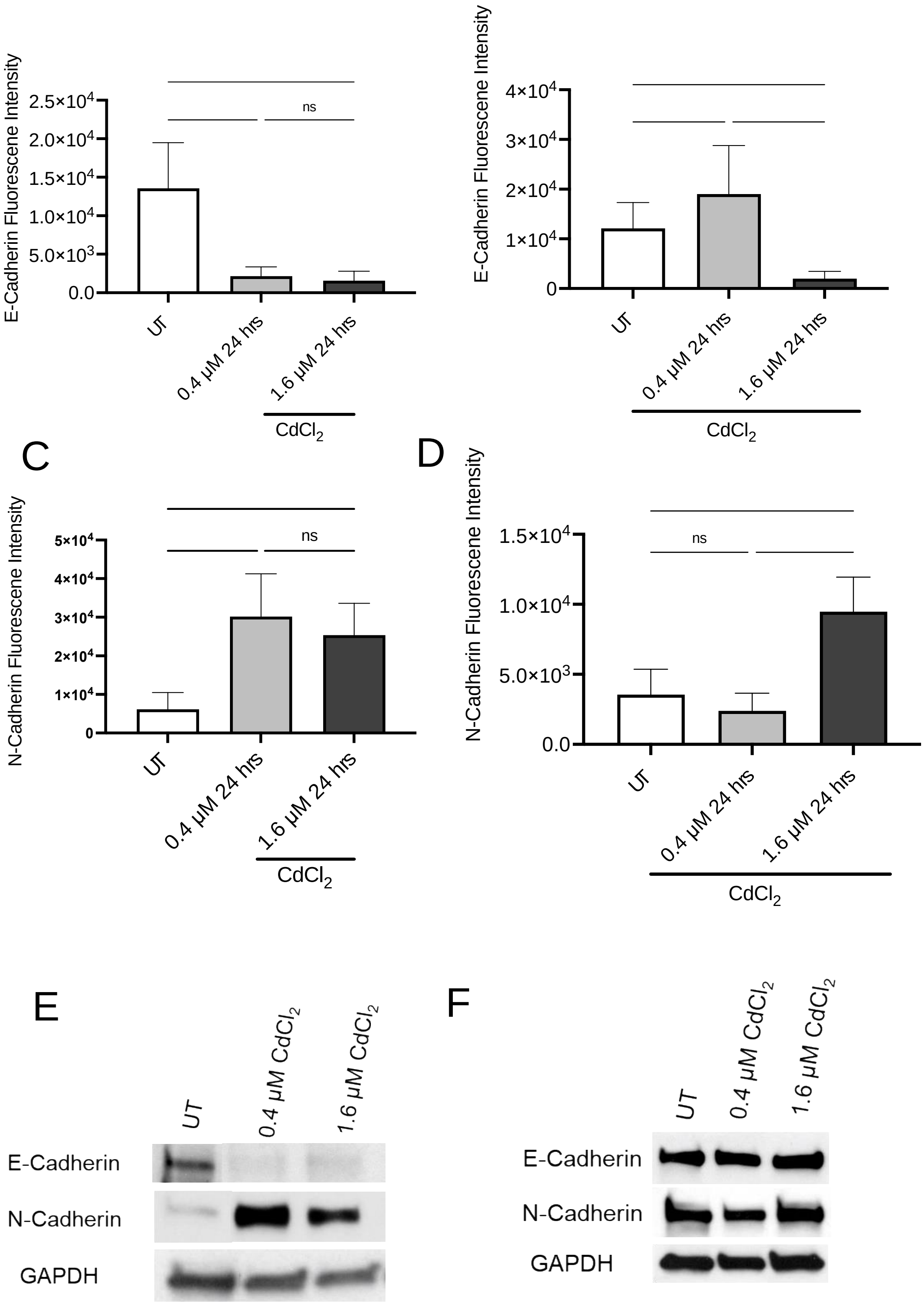

Figure S1. Low doses of Cadmium alter expression in cadherin after 24 h in epithelial cells (**A**,**B**) Fluorescence quantification of at least five random fields from the IF confocal assays for E-cadherin following 24 h treatment of BEAS-2B (**A**) and HEK293 (**B**) cells with 0.4 μM or 1.6 μM Cd. (**C**,**D**) Fluorescence quantification of at least five random fields from the IF confocal assays for N-cadherin following 24 h treatment of BEAS-2B (**C**) and HEK293 (**D**) cells with 0.4 μM or 1.6 μM Cd. Values shown are means ± SE ** $p < 0.01$, **** $p < 0.0001$

vs. UT (Untreated control). (**E**,**F**) Western blot analysis of E-cadherin and N-cadherin from protein samples derived from 24 h treatments with 0.4 μM or 1.6 μM Cd in DMEM.

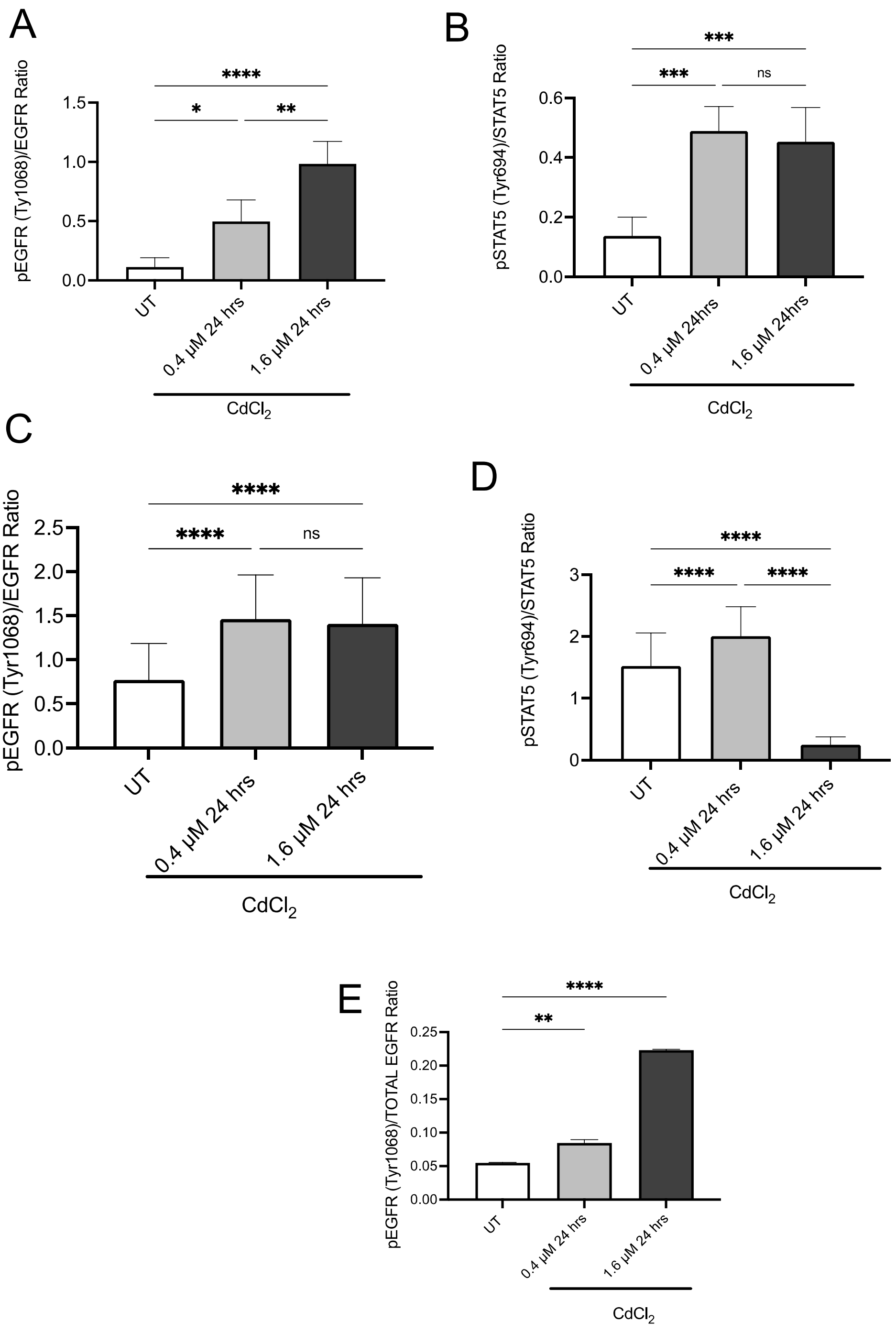

Figure S2. Low doses of Cd increase activation of EGFR/STAT5 signaling on both BEAS-2B and HEK293 cells. (**A**,**C**) EGFR fluorescence quantification of at least five random fields from the BEAS-2B (**A**) and HEK293 (**C**) IF assays, from the 24-hour treatments (Figures 2A and Figure 3A respectively) expressed as fold change (pEGFR over total EGFR ratio). Values shown are means ± SE. **** $p < 0.0001$ vs. UT (Untreated controls), (**B**,**D**) STAT5 fluorescence quantification of at least five random fields from the BEAS-2B (**B**) and HEK293 (**D**) IF assays, from the 24-hour treatments (Figures 2B and Figure 3B respectively) expressed as fold change (pSTAT5 over total STAT5 ratio). Values shown are means ± SE. * $p < 0.05$, ** $p < 0.01$, *** $p < 0.001$,**** $p < 0.0001$ vs. UT (untreated controls) (**E**) EGFR Western blot quantification of Figure 3C, expressed as fold change

(pEGFR over total EGFR ratio). Values shown are means ± SE. ** $p < 0.01$, **** $p < 0.0001$ vs. UT (Untreated controls). (UT = untreated/control).

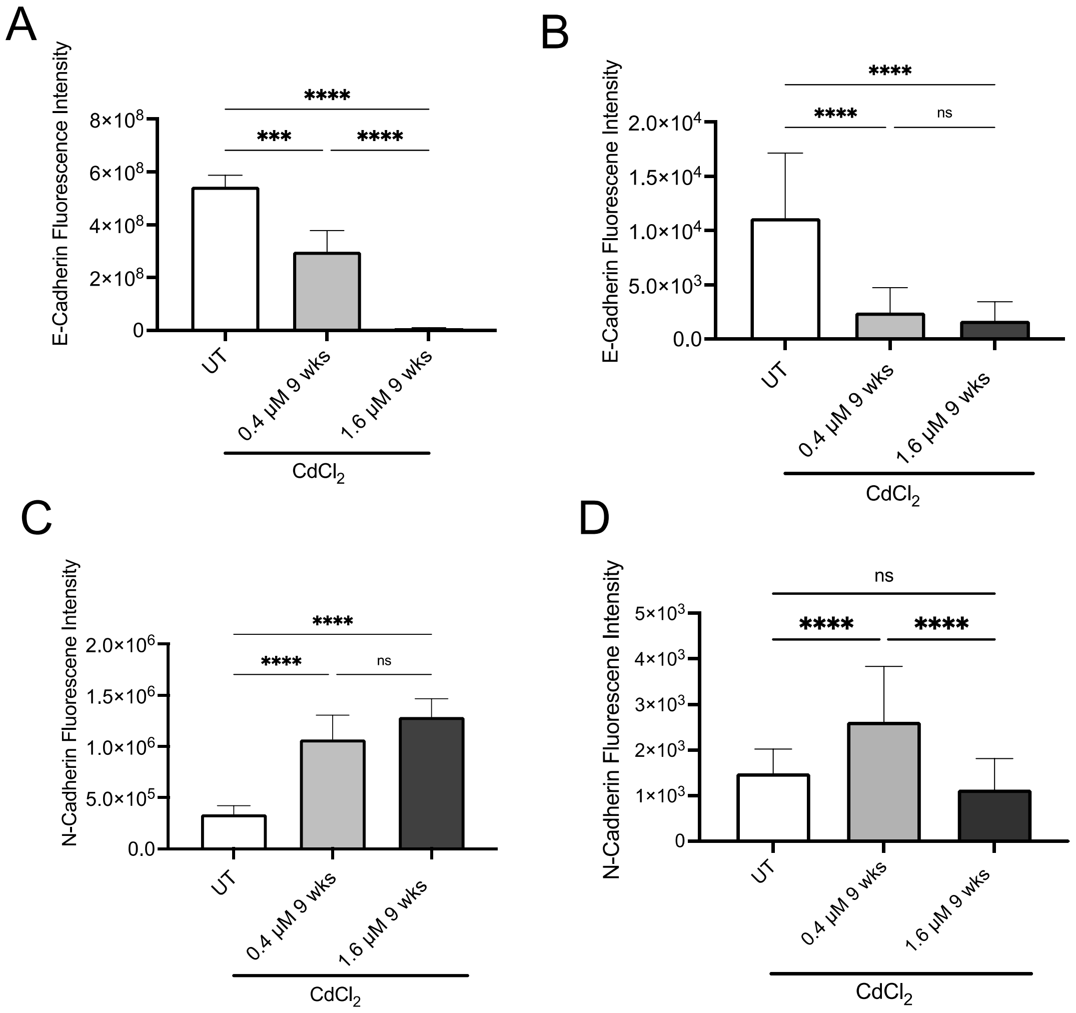

Figure S3. Low doses of Cadmium alter cadherin expression. (**A**,**B**) Fluorescence quantification of at least five random fields from the IF assays for E-cadherin following a 9-week treatment of BEAS-2B (**A**) and HEK293 (**B**) cells with 0.4. μM or 1.6 μM Cd. (**C**,**D**) Fluorescence quantification of at least five random fields from the IF assays for N-cadherin following a 9-week treatment of BEAS-2B (**C**) and HEK293 (**D**) cells with 0.4 μM or 1.6 μM Cd. Values shown are means ± SE. **** $p < 0.0001$ vs. UT (Untreated controls)..

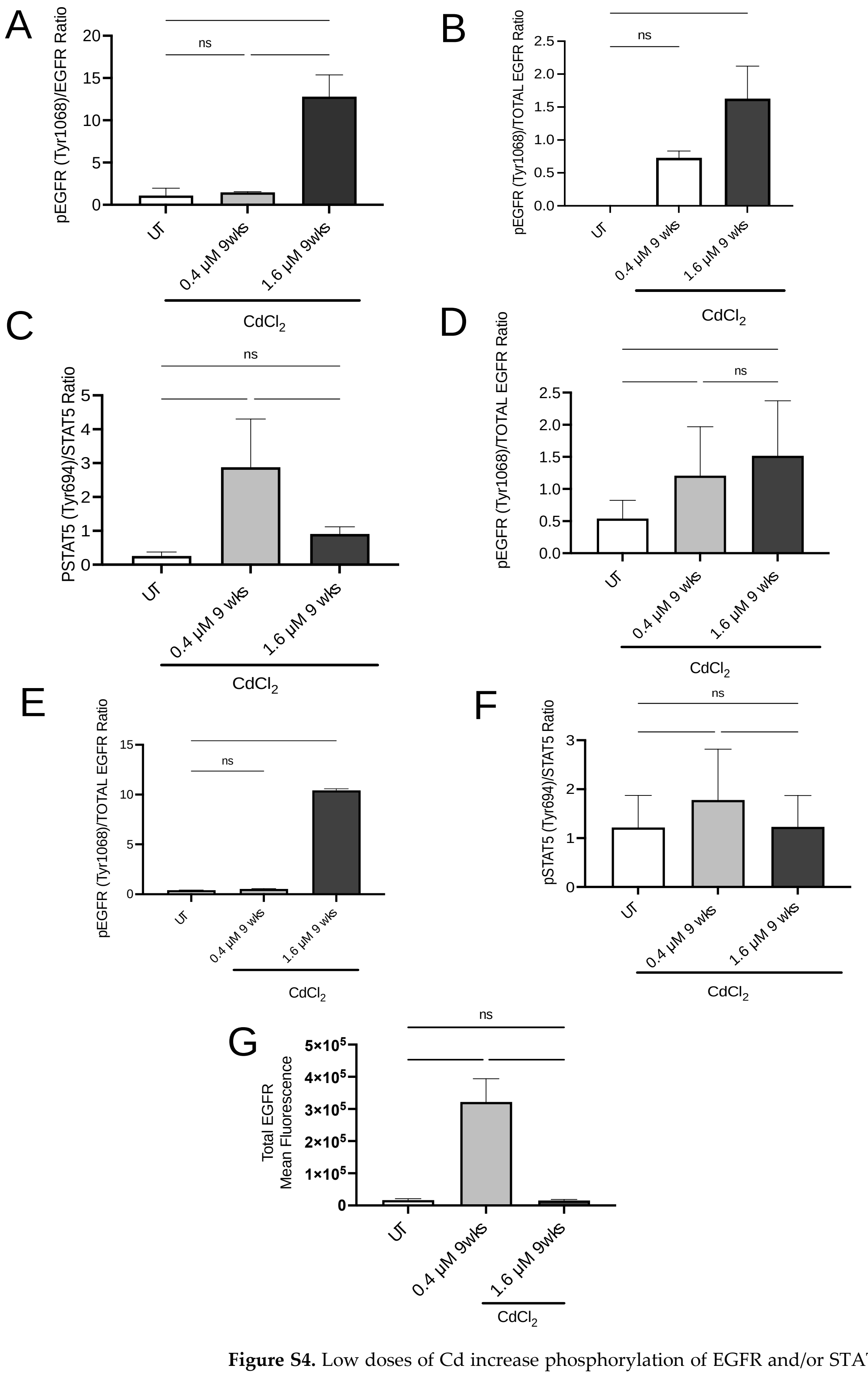

Figure S4. Low doses of Cd increase phosphorylation of EGFR and/or STAT5 but do not activate the EGFR/STAT5 signaling pathway in both BEAS-2B and HEK293 cells. (**A**,**G**,**D**) EGFR fluorescence quantification of at least five random fields from the IF assays for the BEAS-2B (**A**,**G**) and HEK293 (**D**) IF assays, from the 9-week treatments (Figures 5A and Figure 6A, respectively). IF quantification is expressed as fold change (pEGFR over total EGFR ratio) (**A**,**D**),

and mean green (total EGFR) fluorescence (**G**) for the BEAS-2B cells IF assay. (**B**) EGFR Western blot quantification of Figure 5C, expressed as fold change (pEGFR over total EGFR ratio). (**C**,**F**) STAT5 fluorescence quantification of at least five random fields from the IF assays for the BEAS-2B (**C**) and HEK293 (**F**) IF assays, from the 9-week treatments (Figures 5B and Figure 6B respectively) expressed as fold change (pSTAT5 over total STAT5 ratio). (**E**) EGFR Western blot quantification of Figure 6C, expressed as fold change (pEGFR over total EGFR ratio). Values shown are means ± SE. * $p < 0.05$, *** $p < 0.001$,**** $p < 0.0001$ vs. UT (untreated controls).

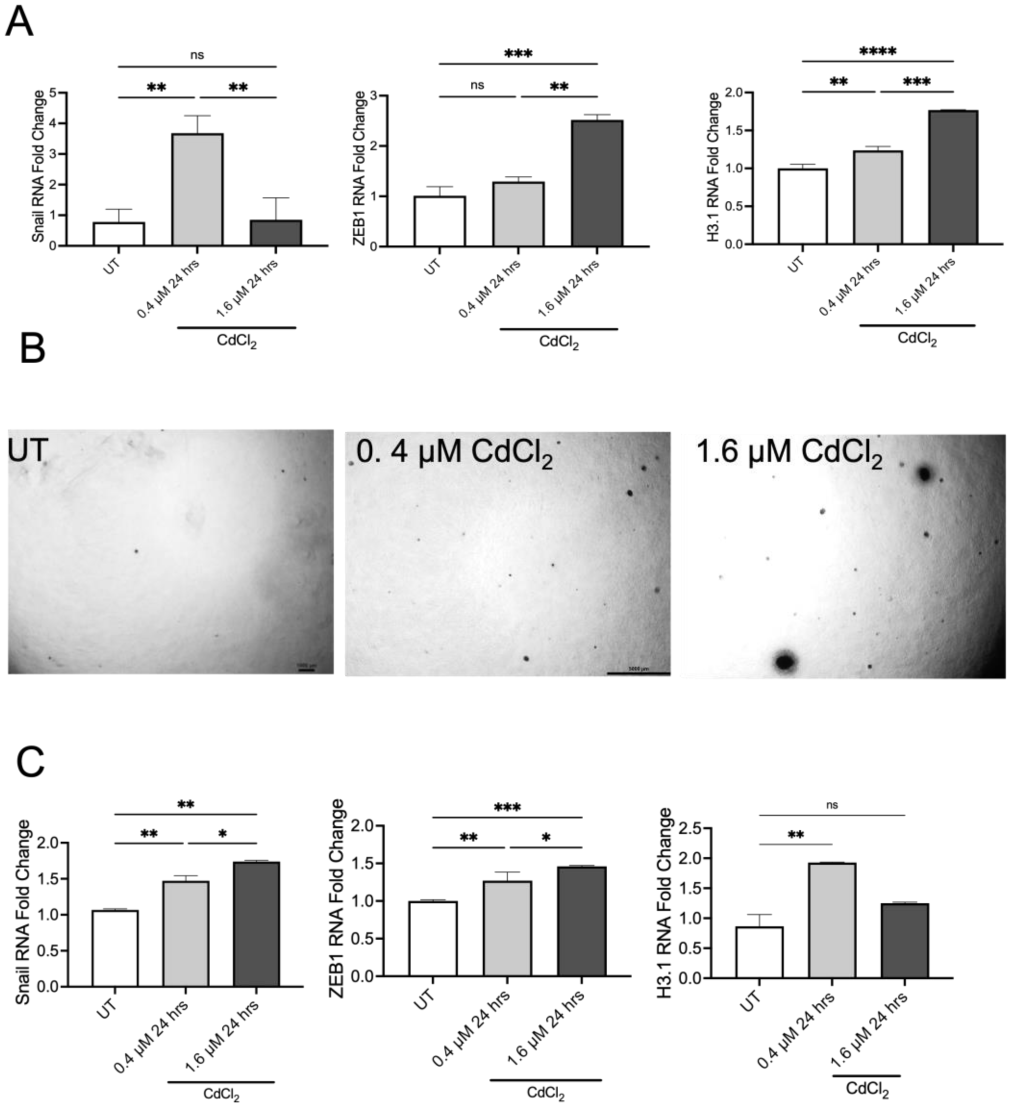

Figure S5. Low doses of Cd induce EMT much sooner in HEK293 cells compared to BEAS-2B cells. (**A**,**C**) mRNA levels of EMT markers from 9-week-old, 24-hour treated HEK293 cells (**A**) and BEAS-2B cells (**B**) with 0.4 μM or 1.6 μM Cd, expressed as fold change. Values shown are means ± SE. * $p < 0.05$, ** $p < 0.01$, *** $p < 0.001$, **** $p < 0.0001$ vs. UT (untreated controls). (**B**) Representative bright-field depicting the clones formed on a soft agar assay to assess anchorage independent growth after exposing HEK293 cells to 0.4 μM or 1.6 μM Cd for 4 weeks. Pictures were taken under 4× magnification.
